# Prevalence of bullying victimisation among primary school children in South Africa: a population-based study

**DOI:** 10.1186/s13104-021-05747-w

**Published:** 2021-08-30

**Authors:** Donnay Manuel, Sabirah Adams, Mulalo Mpilo, Shazly Savahl

**Affiliations:** 1 Centre for Interdisciplinary Studies of Children, Families and Society, University of the Western Cape, Cape Town, South Africa; 2 Language Development Group, Centre for Higher Education Development, University of Cape Town, Cape Town, South Africa

**Keywords:** Bullying victimisation, Children, Prevalence, South Africa

## Abstract

**Objectives:**

Bullying victimisation (BV) among children in South Africa has been identified as a major public health concern. While several studies report on the prevalence rates of BV, there is currently a dearth of research that reports on the prevalence of BV among a national sample of primary school children. This study determines the prevalence rates of BV among a nationally representative sample of school-going children in South Africa across provinces, age, and gender. The sample comprised 7067 children (boys = 45.6%; girls = 54.4%) between the ages of 10–12-years attending 61 primary schools across the nine provincial regions of South Africa.

**Results:**

In terms of ‘being hit’ by other children, percentages range from 22.55% (North West) to 33.34% (Free State). Children in Gauteng (33.59%) and Limpopo (38.54%) had the highest percentage of children being ‘left out’ or excluded. Additionally, across all provinces more than 30% of children reported that they had been ‘called unkind names’. Across gender, boys are more likely to experience all three forms of BV (being hit, left out, and called unkind names). The findings further indicate that 10-year-olds reported being ‘hit’ and ‘left out’, whereas a greater percentage of 12-year-olds reported ‘being called unkind names’ (44.28%).

**Supplementary Information:**

The online version contains supplementary material available at 10.1186/s13104-021-05747-w.

## Introduction

Bullying victimisation (BV) among school children is an international public health concern [1]. It can be understood as a subset of aggressive behaviour [2], or a form of interpersonal aggression [3, 4]. Olweus [5], a pioneer of bullying research, defines bullying as intentionally and repeatedly inflicting harm on an individual, with a power imbalance between the perpetrator and victim. Although there is contestation concerning how bullying is defined, there is a degree of consensus among researchers that in order for bullying to occur there has to be intent, repetitiveness, and a power imbalance [2].

The different forms of bullying have been categorised into two distinct groups, namely direct (including hitting and name-calling) and indirect (including spreading rumours and social exclusion) bullying [6–9]. Research has shown that boys are more likely to engage in direct forms of bullying, whereas girls are more likely to engage in indirect forms of bullying [10–12]. Although the incidence of BV generally decreases with age, some research studies have found an increase in bullying perpetration with age [13, 14] Additionally, there appears to be a shift in the forms of bullying children experience as they age, from direct to indirect forms of bullying [15, 16].

These different conceptualisations and forms of bullying have implications for developing appropriate intervention and policy responses. In particular, it influences the reporting of prevalence rates within and across different contexts. The rate of BV among children is a key indicator of children’s well-being, and an important marker for comparing global social development [17]. Several international studies have reported on the prevalence rates of school-based bullying across various socio-economic contexts. These include the Health Behaviour in School-aged Children (HBSC) conducted across 43 high-income countries in Europe and North America, the Global School-based Student Health Survey (GSHS) conducted across over 90 low- and middle-income countries, the Program for International Student Assessment (PISA), the Progress in International Reading Study (PIRLS), and the Children's Worlds Survey. The prevalence rates in these studies range from 9 to 44.5% [9, 17–23].

A recent international comparative study found that South Africa had the highest prevalence of school-based BV across 15 countries [9]. While earlier studies report prevalence rates between 13 and 43% [22, 24–26], there is currently no study that provides prevalence rates on BV among primary school children using a national representative sample across age groups. The current study addresses this gap in the literature. We use data from Wave 3 of the South Africa Children’s Worlds Study to report on the prevalence rates of BV using a national population-based sample. More specifically, we report on the prevalence of three forms of BV across the nine provinces, gender, and across two age groups (10 and 12). Finally, we also report on the likelihood of children experiencing different forms of BV by province, gender, and age.

## Main text

### Method

#### Research design and sampling

The study uses secondary data from Wave 3 of the South Africa Children’s Worlds: International Survey on Children’s Well-Being (see www.isciweb.org). The survey was conducted across 35 countries, and is the largest multi-national study assessing children’s subjective perceptions of their well-being across different contexts and domains [27]. In South Africa, we used a school-based sample comprising a nationally representative proportionate sample of children aged 10- and 12–years old. In South Africa, children in these two age groups are generally in grades 4 and 6. We used stratified random sampling (proportional allocation), with schools selected proportionate to the number of learners per province for each age group, and stratified further in terms of urban and rural geographical locations. The final dataset consisted of 7067 participants (boys = 45.6%; girls = 54.4%) between the ages of 9 and 12-years (*M*_age_ = 10.79; *SD* = 1.28), in Grades 4 (n = 3383) and 6 (n = 3684), attending 61 primary schools across the nine provincial regions of South Africa.

#### Instrumentation

We translated the questionnaire into seven official South African languages using the International Test Commission’s Guidelines for Adapting and Translating Tests [28]. This process comprised the backward-translation method, the review of an expert panel, and cognitive testing with participants with similar characteristics to the target population. The Children’s Worlds Survey included three items on BV, assessing direct and indirect bullying. These items are as follows:

i. How often in the last month have you been hit by other children in your school?
ii. How often in the last month have you been left out by other children in your school?
iii. How often in the last month have you been called unkind names by other children in your school?

These items were scored on a 4-point frequency scale using the following response options: 0 (never); 1 (once); 2 (2 or 3 times); and 3 (more than 3 times). In the current study, we merged the last two categories (‘2 or 3 times’ and ‘more than 3 times’) into a category called ‘2 or more times’ for ease of interpretation.

#### Data analysis

Data were analysed using Stata 14. The final dataset was weighted based on the proportion of children per province. Weighting is employed with population-level data in order to adjust the sample to be more representative of the target population [29], and to ensure accurate standard errors and parameter estimates [30]. In this descriptive article, we report on the prevalence and frequencies of different forms of BV across age, gender, and the nine provincial regions in South Africa. We also report on the likelihood ratio of experiencing the three forms of BV across the aforementioned groups. We calculated the likelihood ratio by dividing the larger percentage, experiencing BV, with the corresponding smaller percentage of the two groups being compared (age and gender). The ratio represents the likelihood of groups experiencing a type of BV, whereby a larger ratio reflects a greater likelihood/disparity across groups [31].

#### Procedure and ethics

We obtained ethics clearance from the Institutional Review Board of the University of the Western Cape. We obtained consent from both the participants and their parent/guardian. Data collection followed a researcher-administered protocol wherein the research team administered the questionnaire to the participants by reading each question and explaining the response options.

### Results

**Fig. 1:**
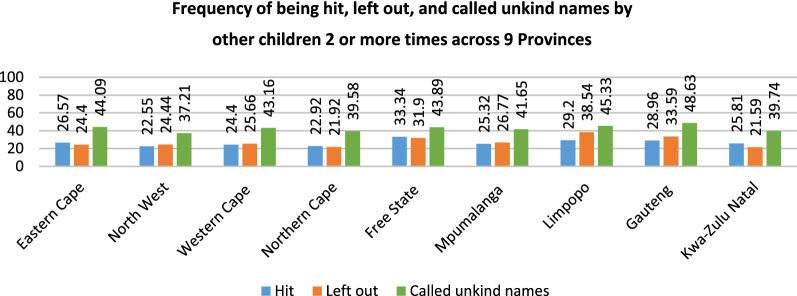
Frequency of being hit, left out, and called unkind names by other children ‘2 or more times’ across nine Provinces

The aim of the study was to determine the prevalence rates of BV among primary school children in South Africa across the nine provinces, age, and gender. We present the frequency of the three BV items across the nine provinces in Fig. 1. We found considerable differences for the different forms of bullying across the provinces. The highest prevalence across all provinces was for being ‘called unkind names’, with the highest prevalence rate in Gauteng (48.63%) and the lowest in North West (37.21%). For those reporting ‘being hit’ two or more times, the prevalence ranged from 22.55% in the North West to 33.34% in the Free State. Gauteng (33.59%) and Limpopo (38.54%) had the highest prevalence of children feeling ‘left out’ (excluded) ‘2-or-more times’ in the last month.

**Fig. 2:**
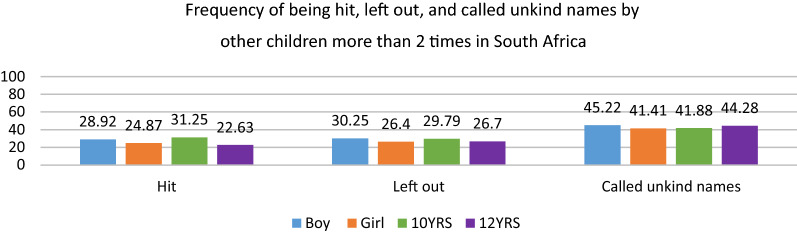
Overall frequency of being hit, left out, and called unkind names by other children ‘2 or more times’ across gender and age

Figure 2 presents the overall frequency of the different forms of BV across gender and age. Boys reported higher prevalence rates across all three forms of BV. A higher proportion of boys reported being hit (28.92%), ‘left out’ (30.25%), and ‘called unkind names’ (45.22%). We also found that a higher percentage of 10-year-olds reported ‘being hit’ (31.25%) and ‘left out’ (29.79%), while a higher percentage of 12-year-olds reported being ‘called unkind names’ (44.28%).

Finally, we present the likelihood ratios of experiencing bullying victimisation across province, age, and gender in Table 1. There were substantial differences in the likelihood ratio among children who experienced different forms of BV across age and gender.

**Table 1:**
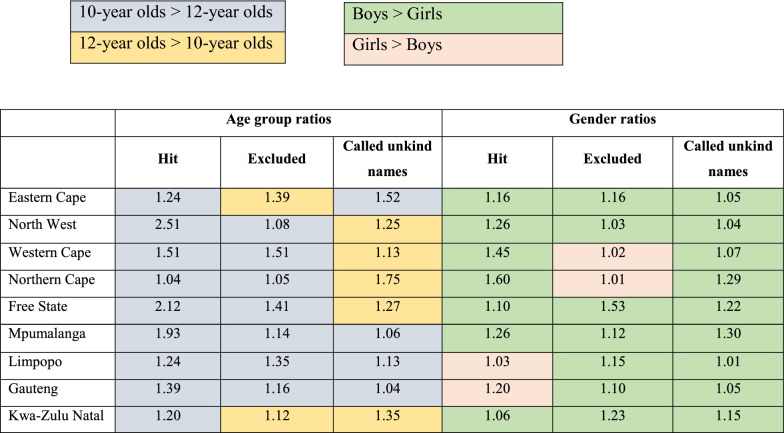
Likelihood ratio of being bullied across age and gender within the nine provinces

We found that 10-year-olds were more likely to be ‘hit’ by other children across all provinces, with the largest group difference in the North West (2.51 times more likely) and the smallest in the Northern Cape (1.04 times more likely). There were similar patterns for being ‘left out’ by other children, with 10-year olds more likely to experience being ‘left out’ in most provinces, with the exception of the Eastern Cape and Kwa-Zulu Natal. We found the largest group difference in the Western Cape with 10-year-olds being 1.51 times more likely to be ‘left out’ than 12-year-olds. However, age differences for being ‘called unkind names’ were less pronounced. In five provinces (North West, Western Cape, Northern Cape, Free Sate, and Kwa-Zulu Natal), 12-year-olds were more likely to be ‘called unkind names’, with the largest difference in the Northern Cape (1.75). On the other hand, in four provinces (Eastern Cape, Mpumalanga, Limpopo, and Gauteng), 10-year-olds were more likely to experience this form of BV (Additional file 1: Table S1).

In terms of gender, boys were more likely than girls to be ‘hit’ in seven provinces; the largest difference was in the Northern Cape with boys 1.60 times more likely than girls to experience this form of bullying. However, in Limpopo (1.03) and Gauteng (1.20), girls were more likely to be ‘hit’ than boys. Similarly, boys were more likely to be ‘left out’ than girls in seven provinces. However, in two provinces (Western and Northern Cape) girls were more likely to be ‘left out’ than boys. Finally, boys were more likely to experience being ‘called unkind names’ in all the provinces, with the greatest gender difference in Mpumalanga (1.30 times more likely), and the smallest (1.04) gender difference in the North West.

### Discussion

The aim of the study was to determine the prevalence rates of three forms of BV among primary school children in South Africa across the nine provinces, across age and gender. Across all provinces, the highest prevalence was for being ‘called unkind names’. This finding aligns to those found in most countries that participated in previous waves of the Children’s Worlds Survey [31]. We found a higher prevalence of BV in the Free State, Limpopo, and Gauteng. There are no obvious reasons explaining this trend, given the diverse geographical (urban and rural) and socio-economic contexts of these provinces. While the Free State and Limpopo provinces have a larger rural population and lower levels of economic productivity, the Gauteng province is largely urban and is the most economically productive province in the country. The prevalence rates were higher for boys than girls across all three forms of BV. This finding aligns to previous cross-cultural research conducted by Savahl et al. [9] and Smith et al. [32]. Younger children experienced being ‘hit’ and ‘left out’ more, while older children were more likely to experience being ‘called unkind names’. This trend is likely as a result of childhood developmental patterns [31, 33].

A trend identified in the empirical literature of boys being more likely to engage in physical forms of bullying and girls more likely to engage in indirect forms of bullying [12, 18] was not found in the current study. Rather, we found that boys were more likely to experience all forms of bullying across most of the provinces. However, while the prevalence was higher for boys than girls, the prevalence rates for girls were similarly high across the three forms of bullying. Consistent with the literature, we found that ‘being hit’ and ‘left out’ decreased with age (see [14, 34]); however, there was no decrease for being ‘called unkind names’. This relational form of bullying appears to have a different trajectory to other forms of bullying and confirms Savahl et al.’s [9] contention that bullying is not a homogenous experience and that there are diverse dynamics across the different forms. Across age, forms of BV also appeared to shift from a physical (being hit) to a verbal form (called unkind names), which is aligned to reported shifts from direct to indirect forms of bullying as children get older [15, 16].

## Limitations

We asked respondents to report on their experiences of three forms of BV over the past month. This restriction may not necessarily capture longer periods of victimisation. The aim of the current study was merely to establish the degree to which children in South Africa experience BV and does not highlight the correlates or predictors of BV. Future studies should therefore focus on the contextual factors associated with BV among children in South Africa.

## Supplementary Information

**Additional file 1**: **Table S1.** Proportion of children per province.

## Data Availability

The data that support the findings of this study are available from the International Society for Child Indicators, but restrictions apply to the availability of these data, which were used under license for the current study, and so are not publicly available. Data are however available from the authors upon reasonable request and with permission of the International Society for Child Indicators.

## Abbreviations

BV: Bullying victimisation
HBSC: Health Behaviour in School-aged Children
GSHS: Global School-based Student Health Survey
PISA: Program for International Student Assessment
UNESCO: United Nations Educational, Scientific and Cultural Organization
PIRLS: Progress in International Reading Study
