# Supplementary material for: Prevalence of bullying victimisation among primary school children in South Africa: a population-based study
Source: BMC Res Notes. 2021 Aug 30;14:342. doi: 10.1186/s13104-021-05747-w (PMC8404275; doi:10.1186/s13104-021-05747-w)
Supplement: Supplementary file 1 — Additional file 1: Table S1. Proportion of children per province. [file 13104_2021_5747_MOESM1_ESM.docx]

**Additional file 1: Table S1.**

**Proportion of children per province**

| **Province** | **% of total registered children per province** | **No. of participants per province** | **Total participants per**  **age group (%)** | | **Gender** | | **% Urban^**^**  **(No. of schools selected)** | **% Rural**  **(No. of schools selected)** |
| --- | --- | --- | --- | --- | --- | --- | --- | --- |
|  |  |  | **10** | **12** | **Boy** | **Girl** |  |  |
| **Western Cape** | 9.15 | 738 | 379 (51.36) | 358 (48.64) | 331 (44.85) | 407 (55.15) | 95.66 (5) | 4.34 (1) |
| **Eastern Cape** | 16.00 | 998 | 475 (47.60) | 523 (52.40) | 526 (52.71) | 742 (47.29) | 35.35 (4) | 64.65 (8) |
| **Limpopo** | 12.69 | 1198 | 640 (53.42) | 558 (46.58) | 571 (47.66) | 627 (52.34) | 10.21 (1) | 89.79 (9) |
| **Free State** | 5.66 | 280 | 130 (46.43) | 150 (53.57) | 122 (43.57) | 158 (56.43) | 82.52 (3) | 17.48 (1) |
| **Gauteng** | 16.78 | 1156 | 497 (42.99) | 659 (57.01) | 517 (44.72) | 639 (55.28) | 97.68 (12) | 2.32 (1) |
| **KwaZulu-Natal** | 21.95 | 1416 | 674 (47.60) | 742 (52.40) | 606 (42.80) | 810 (57.20) | 38.54 (6) | 61.46 (10) |
| **Northern Cape** | 2.43 | 201 | 123 (61.19) | 78 (38.81) | 83 (41.29) | 118 (58.71) | 79.09 (1) | 20.91 (1) |
| **Mpumalanga** | 8.45 | 498 | 231 (46.39) | 267 (53.31) | 228 (45.78) | 270 (54.22) | 36.22 (2) | 63.78 (4) |
| **North West** | 6.88 | 629 | 266 (42.29) | 363 (57.71) | 268 (42.61) | 361 (57.39) | 41.51 (2) | 58.49 (3) |
| **TOTAL** | **100%** | **7114** | **3415 (48.00%)** | **3699 (52.00%)** | **3252 (45.71%)** | **3862 (54.29%)** | **54.88%** | **45.12%** |

**** Urban includes peri-urban and smaller towns**
